# Reduced Specificity of Personal Goals and Explanations for Goal Attainment in Major Depression

**DOI:** 10.1371/journal.pone.0064512

**Published:** 2013-05-15

**Authors:** Joanne M. Dickson, Nicholas J. Moberly

**Affiliations:** 1 Institute of Psychology, Health and Society, University of Liverpool, Liverpool, United Kingdom; 2 Mood Disorders Centre, University of Exeter, Exeter, United Kingdom; Linkoping University, Sweden

## Abstract

**Objectives:**

Overgeneralization has been investigated across many domains of cognitive functioning in major depression, including the imagination of future events. However, it is unknown whether this phenomenon extends to representations of personal goals, which are important in structuring long-term behaviour and providing meaning in life. Furthermore, it is not clear whether depressed individuals provide less specific explanations for and against goal attainment.

**Method:**

Clinically depressed individuals and controls generated personally important approach and avoidance goals, and then generated explanations why they would and would not achieve these goals. Goals and causal explanations were subsequently coded as either specific or general.

**Results:**

Compared to controls, depressed individuals did not generate significantly fewer goals or causal explanations for or against goal attainment. However, compared to controls, depressed individuals generated less specific goals, less specific explanations for approach (but not avoidance) goal attainment, and less specific explanations for goal nonattainment.

**Significance:**

Our results suggest that motivational deficits in depression may stem partly from a reduction in the specificity of personal goal representations and related cognitions that support goal-directed behaviour. Importantly, the findings have the potential to inform the ongoing development of psychotherapeutic approaches in the treatment of depression.

## Introduction

Depression has long been known to be associated with negatively valenced thought content [1]–[2], but investigators have also noted that depression is associated with a tendency towards abstraction and overgeneralization, particularly for negative thought content relating to the self [1], [3]–[5]. Aaron Beck first noted the tendency of depressed patients to overgeneralize single negative events by thinking about their meaning in global terms, often with pessimistic implications for the person [1]. For example, after being involved in a minor traffic accident, a depressed person may conclude that they are completely irresponsible, reckless and blameworthy. Here, we investigate for the first time whether depressed patients' tendency to overgeneralise extends to idiographic motivational constructs in the form of personal goals and the explanations provided for, and against, goal attainment. Because reduced motivation is a principal symptom of depression [6], understanding the specificity of the cognitions relating to personal goals may prove helpful in determining the nature of such deficits.

Early cognitive theorists commented on depressed patients' tendency to think in monolithically negative terms about themselves and to provide overgeneralised, global attributions for negative life events [2]. The tendency to make general (and personal and stable) attributions for uncontrollable events (as in the previous example) is considered to be a vulnerability factor for depression according to the reformulated learned helplessness model and hopelessness theory [7]–[8]. Empirical research indicates that depressed patients do indeed have overgeneral self-attitudes and make global, self-deprecating attributions for negative events [3]–[4]. Depressed persons are also more likely than non-depressed controls to overgeneralize the implications of false negative feedback in laboratory studies [9].

The tendency of depressed persons to overgeneralise extends to the representation of their past selves. Thus, in response to retrieval cues, depressed persons have difficulty recalling memories of specific personal events that took place on a particular day (e.g., ‘the first time I visited the Eiffel Tower’), and instead tend to report broad categories of events that abstract across several episodic memories (e.g., ‘holidays’), relative to controls [5], [10]. This tendency to retrieve ‘overgeneral’ autobiographical memories occurs for both positive and negative retrieval cues, predicts maintenance of depression prospectively, and persists after remission from depressive episode [11]. Furthermore, overgeneral memory recall interacts with stressful life events to predict depressive symptoms in students [12]. A developmental strategy of affect regulation, deficits of executive control, and a tendency to ruminate on abstract themes have all been proposed as possible explanations for the phenomenon, but consensus is lacking on which of these accounts best fits the data [13].

Increasing research suggests that the cognitive processes involved in reconstructing detailed episodic memories are also implicated in the generation of future representations of events involving the self [14]. Consistent with this, there is evidence that the tendency of depressed people to report overgeneral self-representations extends to the imagination of future events that may occur to them. Thus, while depressed individuals generate fewer examples of positive future events (e.g., ‘my best friend's wedding’) than do non-depressed controls [15]–[16], depressed and suicidal people also tend to report future events that are lacking in specific detail, compared to controls [17]. This overgenerality effect for past and future events has also been shown in dysphoric adults [18].

One particularly important representation of the future self is the personal goal construct. Personal goals (e.g., ‘to pass my driving test’) have been defined as internal representations of desired states [19], which are important in organising long-term behaviour and providing meaning in life [20]. Given that episodic details of imagined future selves are more readily generated in the context of goal-related knowledge [21]–[22], the reduced specificity of future events in depression may also be manifested in more abstract representations of personal goals (e.g., ‘gain skills’ rather than ‘obtain an advanced diploma in metalwork’). Although dysregulated goal pursuit has been implicated in the aetiology and maintenance of depression [23], a recent review [24] explicitly suggests that reduced specificity of goal representations is a key marker in clinical depression. However, to date, no research has tested this latter assumption.

Although goal specificity has not previously been investigated by comparing clinically depressed and control groups, undergraduates who described their goal strivings in more abstract (or overgeneral) terms reported more depressive symptoms than those who described their goals in more concrete (or specific) terms [25]. Another study in a non-clinical population found that the personal goals of depressed and mixed anxious-depressed adolescents were less specific than those of non-depressed individuals [26]. Interestingly, this specificity deficit emerged on approach goals representing desirable outcomes (e.g., ‘always be popular*’*) and on avoidance goals representing undesirable outcomes (e.g., ‘avoid becoming unfit’ [27]). These results, which suggest that reduced specificity is not limited to goal content of a particular valence, mirror findings that overgeneral memories are equally prevalent across positive and negative events [11].

A few studies have examined the specificity of goals in clinical populations. Recent suicide attempters generated less specific goals, but not fewer goals, than did hospital controls, although the depression status of these individuals was not known [28]. Recently, Crane and colleagues [29] found that suicidally depressed persons showed increases in goal specificity after mindfulness-based cognitive therapy, compared to persons receiving treatment as usual, and that reduced goal specificity was significantly associated with overgeneral memory, but not with mood improvement. This is consistent with the possibility that depressed persons may differ from controls in the specificity of personal goals, despite findings suggesting that they may not differ on the number of self-reported approach or avoidance goals [30].

Goals are organised in a hierarchy of increasing specificity from general principles to concrete behaviours, and successful self-regulation requires the individual to formulate specific subgoals and plans that advance progress on more abstract goals [31]. Thus, specific goals are crucial for behavioural self-regulation as they provide more direct links to appropriate action [32], whereas overgeneral goal representations may be more ambiguous. Abstract goals may also be associated with less vivid goal attainment imagery, and this may reduce expectancies of success [33] and the extent to which goals generate anticipatory positive affect [34]. In combination, it is likely therefore that reduced expectancies and anticipatory affect will reduce motivation for goal pursuit.

Given that depression has been characterised in terms of a hypoactive approach system and a hyperactive avoidance system [35], one might hypothesise that reduced goal specificity in depression would emerge for approach but not avoidance goals. On the other hand, overgeneral autobiographical memory in depression obtains in response to both positive and negative retrieval cues [11], whereas another study found specificity deficits across approach and avoidance goals in a mixed anxious-depressed school sample [26]. In this study, we made the more conservative prediction that clinical depression will be associated with the generation of less specific goals across approach and avoidance domains, relative to controls.

In order to be sufficiently motivating, goal representations depend on positive outcome expectancies [31], which may themselves be based upon cognitive appraisals including accessible explanations for goal attainment. Not only do the causal explanations people make for negative events play a crucial role in producing depressive hopelessness [7], but the ease with which a person is able to construct reasons for future events is thought to play a crucial role in the subjective probability of an event [33]. Consistent with this, past research [36] has shown that pessimism about future personal events is related to the proportion of reasons generated for why such events may or may not occur. In another study [37], anxious and depressed individuals gave more (‘pro’) reasons to explain why a negative event would occur than (‘con’) reasons to explain why it would not, the relative number of pro versus con reasons was exactly reversed for positive events, and the relative number of pro and con reasons was associated with likelihood judgements for both kinds of event. A recent study of clinically depressed individuals [30] found no significant difference relative to non-depressed controls in the number of reasons generated either for or against goal attainment. Nevertheless, whereas non-depressed individuals generated significantly more pro than con reasons for goal attainment, depressed individuals did not. However, these studies have not examined specificity differences in individuals' causal explanations for goal outcomes, which may be a crucial determinant in facilitating action toward goal achievement, even when goals are more abstract. Reduced specificity of explanations for, and against, goal success may reflect impoverished representations of pathways toward successful goal achievement. Hence, reduced specificity of explanations for goal attainment may be associated with poorer motivation, commitment and effort towards goal attainment. Our second aim is therefore to investigate whether depressed persons' causal explanations for goal attainment are less specific than those reported by controls.

In summary, we hypothesised that depressed adults would generate less specific goals than never-depressed controls, irrespective of whether their goals are focused on approaching rewarding outcomes or avoiding undesirable outcomes. We also hypothesised that depressed adults would generate less specific (‘pro’) reasons for, and less specific (‘con’) reasons against, goal attainment, relative to controls, across approach and avoidance goal outcomes.

## Methods

### Participants

Depressed participants were recruited from NHS Primary Care Teams and Mental Health Trusts in northwest England. The non-depressed participants were recruited from the Primary Care Teams and community in the same region. The Structured Clinical Interview (SCID I) [for Axis I Disorders [38] was administered by trained researchers to assess the presence or absence of current and past major depressive episodes and lifetime psychiatric diagnoses according to Diagnostic and Statistical Manual of Mental Disorders [6] criteria. Inter-rater reliabilities for the trained researchers and clinical psychologist supported the accuracy of these diagnoses (*K*s  = 1). Self-reported depressive symptoms measured by the Beck Depression Inventory (BDI-II [39]) were also used to inform group membership, as described below. In accordance with DSM-IV, exclusion criteria included substance abuse, bipolar disorder, psychotic symptoms, head injury, and mood disorder due to a general medical condition. The depressed group and control group did not differ significantly on age, *t*(42)  = 1.27, *p* = .21, or gender, χ^2^ <1.

#### Depressed group

Twenty-one participants (13 women, 8 men) met DSM-IV criteria for current major depression. Ages ranged from 19 to 74 years (*M* = 37.9, *SD*  = 17.1). Participants reported at least one previous episode of major depression in the past five years. Secondary comorbid anxiety disorders included panic disorder (*n* = 2), social phobia (*n* = 2) and generalized anxiety disorder (*n* = 2). Inclusion criteria also required participants to score in the symptomatic range (>13) on the BDI-II at Time 1 (*M* = 34.1, *SD*  = 11.5) and Time 2 (*M* = 32.3, *SD*  = 11.8).

#### Control group

Twenty-four participants (17 women, 7 men; aged 18 to 81 years, *M* = 31.2, *SD*  = 17.7) had never met criteria for major depression or any psychiatric disorder/Axis I disorder. Inclusion criteria required participants to score in the asymptomatic range (<14) on the BDI-II at Time 1 (*M* = 1.8, *SD*  = 2.2) and Time 2 (*M* = 2.1, *SD*  = 2.6).

### Materials and Procedure

#### Goal Task

The next task comprised two separate, independent measures to assess number of self-generated approach goals and avoidance goals respectively [40]. Prompts were provided to elicit approach goals (‘In the future it will be important for me to…’) and avoidance goals (‘In the future it will be important for me to avoid…’). Participants were instructed to write down specific and discrete goals that they think will typically characterise them at any time in the future (e.g., next week, next month, next year, in a few years), using a separate line for each goal. Participants have 90 s to write down as many personally meaningful and plausible goals that come to mind in each goal condition (approach and avoidance, counterbalanced across participants). We imposed a time limit in each task condition to minimise variations due to task effort. Piloting showed that the time allocation provided in each condition was sufficient for participants to write their response statements, as virtually no responses were given after the 90 s time limit.

#### Goal Explanation Task

The final task comprised two separate, independent measures to assess number of self-generated reasons why participants' two most important approach goals and avoidance goals would (or would not) be achieved [41]. Prompts were used to elicit reasons for (‘pro’) and against (‘con’) goal achievement in each goal condition. Prompts in the approach goal condition were ‘reasons why this would be accomplished?’ (pro reasons) vs. ‘reasons why this would not be accomplished?’ (con reasons). Prompts in the avoidance goal condition were ‘reasons why this would be avoided? (pro reasons) vs. ‘reasons why this would not be avoided?’ (con reasons). Participants were instructed to write down as many plausible, specific, discrete causal explanations that come to mind in each (pro and con) condition for each goal, using a separate line for each reason. Participants are given 90 s in each condition (to help control for differential task effort) and the pro and con tasks were counterbalanced within and across goal conditions. Following this task, participants were thanked and debriefed.

#### Specificity coding of goals and explanations

Two independent judges, both of whom were blind to condition, dichotomously coded all goals as approach vs. avoidance and all reasons as pro vs. con to confirm that participants generated the appropriate goal or reason type in the relevant condition. Inter-rater reliability for these judgements was perfect (*κ*s  = 1).

A dichotomous coding scheme was used to categorise (i) goals and (ii) reasons as either general or specific. We used a binary coding system because we were unable to develop a more finely-grained coding scheme that yielded good inter-rater reliability. A goal was coded as ‘specific’ if it described an explicit aim or target feature and included at least one of the following specific aspects: time, place, or people (e.g., ‘to finish completing the personal development review forms this evening’). A goal was coded as ‘general’ if it referred to a global or abstract aspiration rather than a specific target feature or unique experience (e.g., ‘to be happy’). These criteria were modified slightly to code participants' reasons such that ‘Because I am paying into a pension each week’ would represent a specific reason, whereas ‘Because I try’ would represent a general reason. Inter-rater reliabilities between two independent judges (both blind to group status) for the specificity coding of goals and causal explanations were both good (*K*s>.82).

#### Ethics Statement

The study was approved by the Sponsorship and Registration Committee and the Institute of Psychology, Health and Society at the University of Liverpool. The study also had ethical approval from National Health Service (NHS), the Local Research Ethics Committee (LREC), and NHS Trust Research Governance Committee. Informed written consent was obtained from each participant prior to testing. The study was conducted in accord with the British Psychological Society's ethical guidelines.

#### Statistical Procedures

No data were missing for any of our main dependent variables. Boxplots revealed two participants in the control group who reported high numbers of approach goals, but winsorizing these outlying scores did not change the pattern of significant results, so we included them in the analysis. No outliers emerged for any other variable within each combination of group and goal/reason type. Histograms revealed approximately normally distributed variables within each combination of group and condition, but the proportion of specific goals exhibited marked positive skew. Thus, in addition to the mixed analyses of variance used to test our hypotheses, we conducted nonparametric tests on the proportion of specific goals to confirm significant parametric results.

## Results

### Proportion of Specific Approach and Avoidance Goals

Table 1 presents descriptive statistics for the number and proportion of specific goals generated by each group, illustrating that most goals were coded as general. Although not the focus of this study, there were no significant effects involving group for either the total number of goals generated or goal importance ratings (*p*s>.05).

**Table 1:** Mean (SD) Number and Proportion of Specific Approach and Avoidance Goals by Group.

	Approach			Avoidance		
	Specific	General	% Specific	Specific	General	% Specific
**Depressed**	0.52 (0.75)	5.90 (2.88)	9.6 (14.2)	0.43 (0.68)	4.81 (2.36)	9.6 (15.7)
**Controls**	1.58 (1.41)	4.42 (2.38)	28.4 (27.0)	1.21 (1.02)	3.25 (2.19)	33.7 (32.8)

We submitted proportion of specific goals to a mixed ANOVA with a repeated-measures factor of goal (approach vs. avoidance) and a between-subjects factor of group (depressed vs. control). As predicted, a main effect of group emerged, *F*(1, 43)  = 10.74, *p* = .002, η^2^_p_  = .20, indicating that depressed participants reported less specific goals than non-depressed controls, but there was no significant main effect of goal, nor a goal by group interaction, *F*s<1. Non-parametric Mann-Whitney tests confirmed this significant group difference, revealing that depressed participants were significantly less specific than controls for both approach, *U* = 142.0, *z* = 2.61, *p* = .009, *r* = .39 and avoidance goals, *U* = 135.0, *z* = 2.81, *p* = .004, *r* = .42.

### Proportion of Specific Reasons for and against Goal Attainment

Table 2 presents descriptive statistics for mean number and proportion of specific and general pro and con reasons for approach and avoidance goals. Despite not being the focus of the study, there was no significant effect of group on total number of reasons generated, *p*<.05. Although there was a significant group by reason interaction, *F*(1, 43)  = 7.46, *p* = .009, η^2^_p_  = .15, tests of simple main effects revealed no significant group differences on number of either pro or con reasons (*p*s>.05).

**Table 2:** Mean (SD) Number and Proportion of Specific Pro and Con Reasons for Each Approach and Avoidance Goal.

	Approach			Avoidance		
	Specific	General	% Specific	Specific	General	% Specific
**Pro reasons**						
**Depressed**	0.98 (0.80)	3.31 (1.43)	23.1 (14.8)	1.45 (1.13)	2.50 (1.45)	41.1 (29.6)
**Control**	2.17 (0.92)	2.21 (1.41)	52.5 (25.2)	2.13 (1.06)	2.23 (1.62)	51.6 (25.3)
**Con reasons**						
**Depressed**	1.17 (0.64)	3.05 (2.13)	33.7 (23.4)	1.05 (0.74)	3.07 (1.75)	30.0 (25.3)
**Control**	1.58 (1.02)	1.85 (1.55)	46.0 (29.1)	1.69 (1.03)	1.73 (1.14)	49.8 (24.2)

We conducted a mixed ANOVA on the proportion of specific reasons with repeated-measures factors of goal type (approach vs. avoidance) and reason (pro vs. con) and a between-subjects factor of group (depressed vs. control). A significant main effect of group emerged, *F*(1, 43)  = 10.74, *p* = .002, η^2^_p_  = .20, qualified by a significant three-way group by goal by reason interaction, *F*(1, 43)  = 6.64, *p* = .01, η^2^_p_  = .13. There were no other significant effects. We decomposed the three-way interaction by conducting separate goal by group mixed ANOVAs for mean proportion of specific pro reasons and mean proportion of specific con reasons in turn. The ANOVA on mean proportion of specific pro explanations revealed significant main effects of group, *F*(1, 43)  = 10.32, *p* = .002, η^2^_p_  = .19, and goal type, *F*(1, 43)  = 5.07, *p* = .03, η^2^_p_  = .11, qualified by a significant group by goal interaction, *F*(1, 43)  = 6.18, *p* = .02, η^2^_p_  = .13. Tests of simple effects to decompose the interaction revealed that depressed participants reported proportionately fewer specific pro reasons than controls for approach goals, *F*(1, 70.76)  = 16.29, *p*<.001, but not for avoidance goals, *F*(1, 70.76)  = 2.10, *p* = .15 (see Table 2). The ANOVA on mean proportion of specific con reasons revealed only a significant group effect, *F*(1, 43)  = 5.52, *p* = .02, η^2^_p_  = .11, such that depressed participants reported proportionately fewer specific con reasons (*M* = .32, *SD*  = .22) than controls (*M* = .48, *SD*  = .23) for both approach and avoidance goals.

In sum, the depressed group generated less specific goals across goal types and generated less specific reasons than controls for goal attainment and nonattainment. The only exception was the proportion of specific reasons for successfully avoiding undesired goal outcomes, where no significant group difference emerged. Depressed participants showed a pattern of reduced specificity even though they did not differ significantly from controls on either the number or importance of goals and reasons, so the results cannot be explained in terms of a fluency deficit.

## Discussion

To our knowledge, this is the first investigation of the specificity of goals and the specificity of reasons for and against goal attainment generated by depressed individuals. As predicted, compared to non-depressed controls, depressed individuals reported goals that were less specific. Furthermore, depressed individuals gave less specific reasons for and against attainment of approach goals than controls, and less specific reasons against (but not for) attainment of avoidance goals. This qualitative difference emerged despite the absence of a significant group difference in goal importance ratings. Notwithstanding the limitations imposed by our correlational design, our results suggest that motivational dysfunction in depression [6] may be underpinned by impoverished cognitive representations of goals. Our findings indicate that the tendency towards overgeneralisation in depression extends from episodic representations of the past and future self to representations of personal goals, for both desirable and undesirable outcomes. This is not unexpected given that goals are thought to derive much of their motivational impetus from specific autobiographical memories [42], while current goals have been argued to support the construction of specific autobiographical memories [43].

Control theory suggests that goals may be construed at various levels of abstraction, from abstract principles to concrete actions [31]. According to this account, a person's ability to shift mentally between abstract and concrete representations of goals is crucial for effective self-regulation. Construing goals at abstract levels (e.g., ‘be a skilful person’) without the ability to construe them in more concrete ways (e.g., ‘practise the piano for one hour every evening’) may make it difficult to pursue goals effectively and render self-regulation more difficult because goal pursuits need to be translated into concrete behavioural tendencies. Setting specific goals is one factor that improves self-regulatory performance [32]. Our results support the notion that an inflexibly abstract construal of personal goals may underlie depression, further research is required to ascertain its status as a transdiagnostic marker (e.g., bipolar disorder, generalised anxiety disorder [24]). Future investigations could also examine whether goal abstraction serves as a vulnerability factor for depression in never-depressed individuals.

Reduced specificity of goals and explanations is likely to undermine motivation toward goal attainment and impede the formation of a coherent sense of self [20]. Inflexibly abstract goal representations may have other detrimental effects. For example, it has been suggested that individuals who construe their goals more abstractly place greater value on the importance of their goals rather than goal attainment whereas individuals who construe their goals in concrete forms tend to be more focused on the process of goal attainment itself [44]. Self-regulation at an inflexibly abstract level may increase vulnerability to sad mood because minor setbacks are perceived as impediments to the core values of the self. Furthermore, depressed individuals may find it difficult to disengage from unachievable higher-order goal pursuits that they perceive as personally important, even when failing to make goal progress. In the face of repeated goal attainment failures, disengagement may be adaptive [45]. Although failure to disengage from unsuccessful goal pursuits would be expected to instigate rumination [46], this is especially likely when goals are represented at an abstract level [47]. Furthermore, the predominance of abstract representations may foster a more abstract form of rumination, which has been suggested to be a dysfunctional characteristic of depressive thinking [48]. Abstract rumination about goal pursuit may then make overgeneral representation of events more likely, feeding a vicious cycle.

We also found evidence for reduced specificity of causal explanations for goal attainment in our depressed sample. The ability to generate specific explanations for goal attainment is likely to be an essential determinant in motivating goal-directed behaviour, because the person can more readily simulate the desired outcome [33]. These simulations increase expectancies of success, which then increase motivation [31]. Specific explanations may even mitigate the self-regulatory challenges of abstract goals. For example, a general goal ‘to be happy’ may be realised if a person can identify specific reasons to explain why their goal will be achieved (e.g., ‘because I have close supportive friends that I socialise with each week’). For the most part, however, our results showed that depressed individuals had a propensity to generate abstract explanations for and against goal attainment and these may themselves reinforce abstract goal representations. The capacity to define specific reasons for, and against, goal success is arguably a more cognitively taxing task for abstract than for specific goals. A related possibility is that reduced specificity of causal explanations may undermine a person's ability to learn from goal success and failure, hindering successful self-regulation. Although our results revealed that depressed and never-depressed individuals did not differ significantly in their ability to generate specific reasons for attaining avoidance goals, we believe that interpretation of this finding should await replication.

It is possible that reduced specificity for goals and explanations may be a consequence of impaired executive functioning, which limits the ability to generate personal goal details. Although depressed individuals generated as many goals as never-depressed individuals, it is likely that generation of specific detail requires more executive resources than simply listing desired and undesired outcomes, so future research should seek associations between measures of executive functioning and goal specificity [49]. Theoretical explanations for the association between overgeneral autobiographical memory and psychopathology [11], [13] have implicated abstract rumination and reduced executive function, and our results suggest that these factors might also extend to overgeneral personal goals and causal explanations in depression. The suggestion from this literature that overgeneral memory may develop as a consequence of an affect regulation strategy could also be relevant for our findings. It might therefore be fruitful to investigate whether people who have met with childhood adversity construe their goals in more abstract terms to avoid intense negative emotions that might accompany autobiographical memories that specific goal representations may cue [50].

Our findings clearly implicate reduced specificity of personal goals and goal explanations in the psychopathology of depression. Past experimental research suggests that processing style manipulations affect cognitive processes. For instance, concreteness training that aims to reduce overgeneral thinking has been shown to reduce dysphoria [51]. Research also suggests that motivational interventions emphasising goal concreteness, among other aspects of goal functioning, improve well-being in a non-clinical population [52]. Psychological therapies could do well to address the overgeneralisation of personal goals and reasons as a psychological deficit in depression. For instance, research suggests that mindfulness-based cognitive therapy for chronic depression enhances the specificity of life goals and increases expectancies for goal attainment post-therapy [29]. Psychological therapies aimed at assisting individuals to set specific goals and to formulate specific reasons for goal achievement may increase motivation for goal attainment and promote adaptive self-regulation and well-being.

Some methodological considerations of the present study deserve comment. First, participants were required to list brief, single-statement responses, which may partly account for the relatively high proportion of general responses reported. Second, in contrast to previous non-clinical studies that have examined goal specificity using three graded categories [26], we used only two coding categories. Therefore our specificity measure was relatively coarse, although it was still sufficiently sensitive to detect reliable group differences. Finally, our lack of a non-depressed psychiatric comparison group means that it is unclear whether the pattern of reduced specificity for goals and causal explanations is limited to clinical depression, or would also be found in other psychiatric conditions, reflecting a transdiagnostic marker [24].

Despite the important role of personal goals in human experience, depression has rarely been investigated from this perspective. To our knowledge, this is the first study to investigate reduced specificity of goals and accompanying causal explanations in clinical depression. Our results suggest that difficulties representing specific goals and causal explanations for goal attainment and nonattainment are implicated in the psychopathology of depression. We propose that these reduced levels of specificity may be consequential for reduced levels of motivation and impaired self-regulation. Though the implications of these results are currently limited by the cross-sectional design, further research on the specificity of motivational representations has the potential to inform the development of effective clinical treatments for depression.
